# Identification of α-Amyrin 28-Carboxylase and Glycosyltransferase From *Ilex asprella* and Production of Ursolic Acid 28-*O*-β-D-Glucopyranoside in Engineered Yeast

**DOI:** 10.3389/fpls.2020.00612

**Published:** 2020-05-20

**Authors:** Xiaoyu Ji, Shumin Lin, Yuanyuan Chen, Jiawei Liu, Xiaoyun Yun, Tiancheng Wang, Jialiang Qin, Chaoquan Luo, Kui Wang, Zhongxiang Zhao, Ruoting Zhan, Hui Xu

**Affiliations:** ^1^Research Center of Chinese Herbal Resource Science and Engineering, Guangzhou University of Chinese Medicine, Guangzhou, China; ^2^Key Laboratory of Chinese Medicinal Resource from Lingnan (Guangzhou University of Chinese Medicine), Ministry of Education, Guangzhou, China; ^3^Joint Laboratory of National Engineering Research Center for the Pharmaceutics of Traditional Chinese Medicines, Guangzhou, China; ^4^Department of Pharmacology, Shantou University Medical College, Shantou, China; ^5^School of Pharmaceutical Sciences, Guangzhou University of Chinese Medicine, Guangzhou, China

**Keywords:** *Ilex asprella*, Triterpenoid saponins, Biosynthesis, CYP, UGT

## Abstract

*Ilex asprella* is a medicinal plant that is used extensively in southern China. The plant contains ursane-type triterpenoids and triterpenoid saponins which are known to be responsible for its pharmacological activities. Previously, a transcriptomic analysis of *I. asprella* was carried out and the gene *IaAS*1, which is important in the formation of the core structure α-amyrin, was identified. However, the genes related to the subsequent derivatization of the core structures of the triterpenoid remain largely unknown. Herein, we describe the cloning and functional characterization of an amyrin 28-carboxylase IaAO1 (designated as IaCYP716A210) and a glycosyltransferase IaAU1 (designated as UGT74AG5), based on transcriptomic data. The expression of *IaAO*1 in an α-amyrin producing yeast strain led to the accumulation of ursolic acid. An enzyme assay using recombinant protein IaAU1 purified from *E. coli* revealed that IaAU1 can catalyze the conversion of ursolic acid to ursolic acid 28-*O*-β-D-glucopyranoside. IaAU1 has regiospecificity for catalyzing the 28-*O*-glucosylation of ursane-/oleanane-type triterpene acids, as it can also catalyze the conversion of oleanolic acid, hederagenin, and ilexgenin A to their corresponding glycosyl compounds. Moreover, co-expression of *IaAO*1 and *IaAU*1 in the α-amyrin-producing yeast strain led to the production of ursolic acid 28-*O*-β-D-glucopyranoside, although in relatively low amounts. Our study reveals that *IaAO*1 and *IaAU*1 might play a role in the biosynthesis of pentacyclic triterpenoid saponins in *I. asprella* and provides insights into the potential application of metabolic engineering to produce ursane-type triterpene glycosides.

## Introduction

Triterpenoids and triterpenoid glycosides constitute a major class of plant secondary metabolites, which are thought to be involved in defense against pathogens and pests (Singh and Sharma, 2015). Compounds such as ginsenosides and glycyrrhizic acid have also been shown to possess health benefits in humans (Kim et al., 2017). However, access to these compounds is limited due to their low levels in plants and difficulties in their purification and chemical synthesis. Unraveling the biosynthetic pathways used for their production might provide the possibility of improving their availability through synthetic biology (Zhou et al., 2015).

Over the past several decades, tremendous interest and progress in the understanding of the biosynthesis of triterpenoids and triterpenoid glycosides have been observed. Generally, triterpenoid glycoside is assembled from six isoprene units followed by cyclization and scaffold modifications. The cyclization reaction mediated by oxidosqualene cyclases (OSCs) is the first diversifying step in the biosynthetic pathway. So far, more than 100 triterpene scaffolds have been reported, primarily including lupane, dammarane, oleanane (derived from β-amyrin), and ursane (derived from α-amyrin) (Shang and Huang, 2019). Recently, a novel triterpene orysatinol was identified, which even widen the potential scape of triterpene scaffolds that could exist in nature (Xue et al., 2018; Stephenson et al., 2019). The subsequent site-specific oxidation and glycosylation of the cyclic scaffold are catalyzed by cytochrome P450 monooxygenases (CYPs) and UDP-dependent glycosyltransferases (UGTs), respectively, conferring further structural and functional diversity.

Both CYPs and UGTs belong to multigene families and are involved in numerous metabolic processes including those related to triterpenoid saponins. To date, several plant CYPs have been functionally characterized and their diverse roles in triterpene scaffold modification have been reviewed (Miettinen et al., 2017). Members from different classes (e.g., CYP51, CYP71, CYP72, CYP85) have been shown to be associated with triterpene scaffold oxidation. Moreover, the reactions catalyzed by CYPs are extremely diverse, including desaturation, oxidation, and C-C bond cleavage. In the case of UGTs, a few enzymes have been identified, including members within the UGT71, UGT73, UGT74, UGT85, UGT91, and UGT94 families (Rahimi et al., 2019). They catalyze versatile glycosylation reactions that result in variations in the number of sugar chains, composition, and position on the triterpene scaffold (Seki et al., 2015; Xu et al., 2016; de Costa et al., 2017; He et al., 2018). Considering the huge diversity of triterpene-related CYPs and UGTs, as well as the fact that the majority of plant triterpene compounds are biosynthesized in species-specific manner, it is of interest to isolate and characterize more triterpene tailoring enzymes CYPs and UGTs from various plant species to extend our knowledge of triterpene metabolism and for utilization of these enzymes.

**FIGURE 1:**
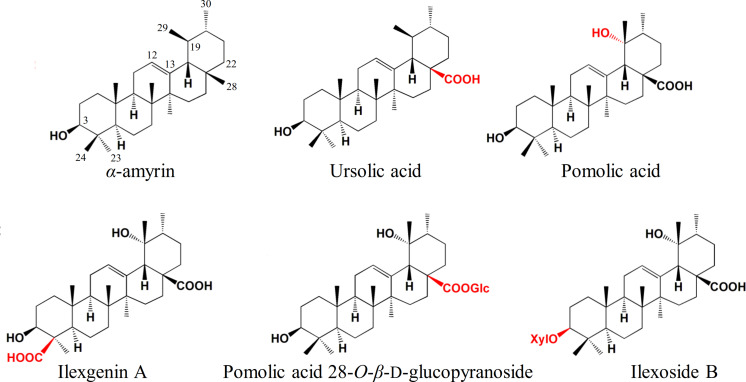
Ursane-type Ilex triterpenoids derived from multiple modifications of α-amyrin. Compounds were depicted to demonstrate the commonly oxidation and glycosylation sites in *I. asprella*. Substituents and substitution positions were indicated in red.

*Ilex asprella* is a medicinal plant that originates from southern China and its root is usually used to treat influenza and pharyngitis. *I. asprella* contains a wide range of triterpenoids and related saponins which possess various bioactivities such as anti-inflammatory, anticancer, and antiviral activities. Most Ilex triterpenoids are of the ursane-type and are derived from multiple modifications of α-amyrin (Figure 1). Oxidative modification occurs most commonly at positions C-19, C-24, and C-28 of the ursane skeleton, while glycosylation occurs at positions C-3 and C-28 (Zhou et al., 2012; Peng et al., 2016; Wen et al., 2017). Although the pharmaceutical and physiological effects of these triterpenoids and related saponins are well-known, our understanding of their biosynthesis in *Ilex asprella* remains limited.

Previously, we have obtained the transcriptome of *I. asprella* using RNA-sequencing (GenBank accession number SRP035767). Analysis of the transcriptome revealed several OSC, CYP, and UGT genes that could be potentially involved in triterpenoid biosynthesis pathway. Among these, two triterpene cyclases, *IaAS*1 and *IaAS*2, which catalyze the cyclization of 2,3-oxidosqualene to form α- and β-amyrin in different ratios, have been identified (Zheng et al., 2015). However, the enzymes involved in the oxidation and glycosylation steps of triterpene biosynthesis in *I. asprella* remain largely unknown. In this study, we report the identification of IaAO1 (named as IaCYP716A210) which can catalyze the C-28 carboxylation of α-amyrin, and a UDP-glycosyltransferase IaAU1 (named as UGT74AG5) that has regiospecificity for catalyzing the 28-*O*-glucosylation of ursane-/oleanane-type triterpene acid. Furthermore, we successfully co-expressed *IaAO*1 and *IaAU*1 in yeast carrying *IaAS*1, resulting in the production of an unusual glycoside ursolic acid 28-*O*-β-D-glucopyranoside.

## Materials and Methods

### Sequence Analysis

Complete amino acid sequences of CYPs and UGTs known were collected from NCBI^1^ for analysis. Multiple sequence alignments were performed using the software Clustal Omega^2^. The phylogenetic tree was constructed using the maximum likelihood method with Molecular Evolutionary Genetics Analysis Program (MEGA7.0) (Sudhir et al., 2016). A bootstrap analysis with 1,000 replicates was used to assess the strength of the nodes in the tree (Felsenstein, 1985).

### cDNA Preparation and Cloning of *IaAO*1 and *IaAU*1

Total RNA was extracted from the leaves of 2-year-old *I. asprella* using a HiPure Plant RNA Mini Kit (Magen, Guangzhou, China). The polyadenylated RNA was reverse transcribed into cDNA using a TransScript II All-in-One First-Strand cDNA Synthesis SuperMix (TransGen Biotech, Beijing, China) according to the manufacturer’s protocol. Using cDNA as the template, the coding regions of *IaAO*1 and *IaAU*1 were amplified using the Primer STAR high-fidelity DNA polymerase (Takara, Dalian, China). The PCR products obtained were purified and ligated into the vector *pEASY*-T5 and subsequently recombinant plasmids were used to transform *E. coli Trans*1-T1 competent cells using a *pEASY*-T5 Zero Cloning Kit (TransGen Biotech, Beijing, China). Both of the recombinant plasmids were verified by sequencing. All the sequences of the primers used in this study are shown in Supplementary Table S1. Detail information about strains and plasmids is listed in Supplementary Table S2.

### Heterologous Expression of *IaAO*1 in Yeast

Two pairs of In-Fusion primers were designed based on the sequences of *IaAO*1 and the yeast expression vector pESC-TRP. After amplification, the coding region of *IaAO*1 was ligated into the vector pESC-TRP at the *Eco*RI and *Spe*I sites using the In-Fusion HD Cloning Kit (Takara, Dalian, China). The plasmid obtained was named pT*IaAO1*. Using a standard lithium acetate protocol, pT*IaAO1* was transformed into an α-amyrin-producing strain of *Saccharomyces cerevisiae* WAT11tfAX (a WAT11-derived yeast strain with an integration of *IaAS*1 in the genome created using CRISPR/Cas9, unpublished data). After 16-h of growth in SC-T media containing 2% glucose at 30°C, the transformed yeast cells were washed three times with sterile water, re-suspended in SC-T media containing 2% galactose and allowed to grow for 48 h. An equivalent number of yeast cells were harvested at different time points and extracted for total protein. The target protein was identified using an anti-His mouse monoclonal antibody (TransGen Biotech, Beijing, China) by western blotting. Yeast transformed with the empty vector pESC-TRP was used as a negative control. The positive transformants were selected and incubated for 7-day. Cell metabolites were extracted, derivatized and analyzed by GC-MS according to the method established previously (Qin et al., 2019).

### Expression and Purification of Recombinant IaAU1

Similarly, *IaAU*1 was ligated into the *Escherichia coli* expression vector pET32a (+) at the *Eco*RV and *Sac*I sites using In-Fusion cloning. The pET-*IaAU1* plasmid obtained was used to transform *E. coli* Rosetta (DE3) cells. Transformants were cultured in LB media with appropriate antibiotics and induced with 0.1 mM IPTG. After harvesting the cells, total proteins were extracted and the recombinant protein was purified by Ni^2+^-NTA chromatography (Qiagen, Germany). SDS-PAGE was performed to assess the expression levels and purity of the recombinant IaAU1.

### IaAU1 Enzyme Assay

IaAU1 activity was measured in a final volume of 200 μL of a buffer consisting of 50 mM Tris (pH 7.5), 10 mM MgCl_2_, 1 mM DTT, 20–60 μg purified IaAU1, 250 μM sugar donor UDP-glucose (UDP-Glc), and 1 mM sugar acceptor (Meesapyodsuk et al., 2007; Naoumkina et al., 2010; de Costa et al., 2017). Six different triterpene sapogenins, ursolic acid (Urs), ilexgenin A (Ilex), oleanolic acid (Ole), hederagenin (Hed), glycyrrhetic acid (Gly), and soyasapogenol B (Soy), were used as sugar acceptors. The reaction was carried out at 30°C for 30 min, and then stopped by adding two volumes of ethyl acetate. The ethyl acetate phase was removed, evaporated to a volume of about 100 μL and analyzed by thin-layer chromatography (TLC). The TLC plate was developed using C_6_H_14_:CH_3_COOC_2_H_5_:CH_3_COOH (1:12:0.5) as the mobile phase and visualized by spraying with 20% sulfuric acid in absolute ethyl alcohol followed by heating at 105°C for 3 min. The exact mass to charge ratio (*m/z*) was measured with a high-resolution mass spectrometer (Orbitrap Fusion^TM^ Tribird^TM^, Thermo Fisher, San Jose, CA, United States). Furthermore, the assay with Urs as the sugar acceptor was carried out at a preparative scale and the product generated was isolated using a Sephadex^TM^ LH-20 column. The purified product was structurally characterized using MS and NMR analyses.

### Co-expression of *IaAO*1 and *IaAU*1 in Yeast

Two separate yeast strains carrying both *IaAO*1 and *IaAU*1 genes were constructed. First, *IaAU*1 was ligated into the yeast expression vector pESC-URA using In-Fusion Cloning. The resulting plasmid pU*IaAU1* was used to transform the abovementioned yeast strain, WAT11tfAX expressing IaAO1, to produce the strain WAT11S1. Alternatively, *IaAO*1 and *IaAU*1 were sub-cloned into the yeast expression vector p426GPD to create the expression cassettes *P*_GAP_-*IaAO*1-*T*_CYC__1_ and *P*_GAP_-*IaAU*1-*T*_CYC__1_, respectively. The *P*_GAP_-*IaAO*1-*T*_CYC__1_ cassette was then integrated into the *ade2* locus of strain WAT11tfAX via CRISPR/Cas9 while *P*_GAP_-*IaAU*1-*T*_CYC__1_ was subsequently integrated into the *bts*1 locus, resulting in strain WAT11S2. WAT11S1 and WAT11S2 were cultured in SC-U-T and YPD media, respectively, at 30°C for 7 days. The cells were then harvested for metabolite extraction. Cell pellets were suspended in 20 mL of sterile water and disrupted using a high pressure homogenizer (20,000 psi, 50 s) (D-6L, PHD Technology LCC, Saint Paul, MN, United States). After extraction with the same volume of *n*-butanol twice, the organic phases were concentrated by evaporation and the residues were reconstituted in methanol and subjected to an LC-MS/MS analysis.

### LC-MS/MS Analysis

HPLC separation was performed using a Surveyor plus HPLC system (Thermo Fisher, Singapore) equipped with a Hypersil Golden C_18_ column (100 mm × 2.1 mm, 3 μm particles) at 20°C. The mobile phase consisted of water (component A) and methanol (component B) and the flow rate was 0.2 mL min^–1^. The gradient program was: 0–2.0 min, 30% B; 2.0–5.0 min, 30–80% B; 5.0–13.0 min, 80% B; 13.0–15.0 min, 80–90% B; 15.0–25.0 min, 90% B. HESI-MS/MS data were acquired with a triple quadrupole mass spectrometer (TSQ Quantum Access, Thermo Fisher, United States) coupled with an electrospray source in positive ionization mode. The MS parameters were set as follows: vaporizer temperature 375°C, capillary temperature 300°C, sheath gas 40 (arbitrary units), aux gas 5 (arbitrary units) and spray voltage 3.5 kV. For qualitative and semi-quantitative analysis of metabolites, selected reaction monitoring (SRM) mode was used. The contents of ursolic acid 28-*O*-β-D-glucopyranoside were determined by calculating relative peak areas using the product prepared from IaAU1 enzymatic activity assay as standard.

## Results

### Screening of the Candidate Genes *IaAO*1 and *IaAU*1 for Triterpene Oxidation and Glycosylation at C-28

**FIGURE 2:**
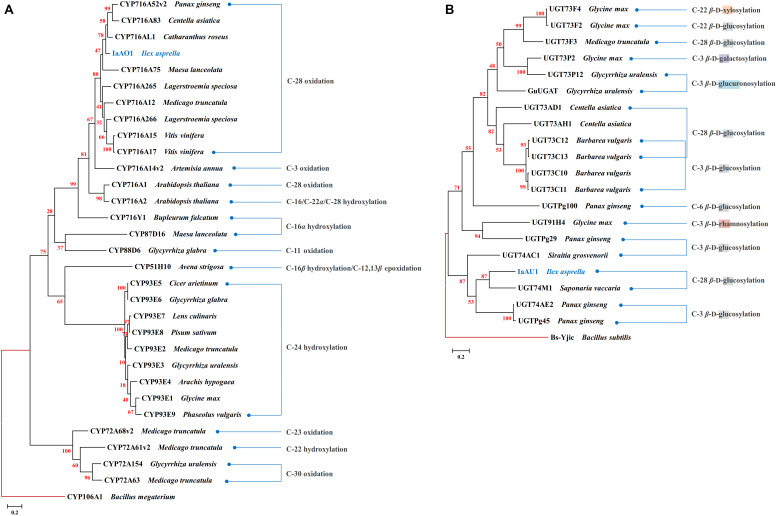
Phylogenetic analysis of IaAO1 **(A)** and IaAU1 **(B)** with known triterpene CYPs and UGTs, respectively. Protein sequences of randomly selected triterpene CYPs and UGTs were retrieved from NCBI. Genes isolated in this study were colored in blue. Red branches represented outgroup. Scale bar indicated the number of amino acid substitutions. GenBank Accession Numbers of the proteins are given in Supplementary Material.

In the transcriptomic data of *I. asprella*, more than 200 transcripts were annotated as CYPs. A phylogenetic analysis revealed a putative CYP gene (*IaAO*1) that was closely clustered with CYP716AL1 (Huang et al., 2012), which was identified from *Catharanthus roseus* as a multifunctional C-28 oxidase capable of converting α-amyrin, β-amyrin, and lupeol to ursolic, oleanolic, and betulinic acids, respectively (Figure 2A). Sequence analysis of *IaAO*1 revealed an open reading frame of 1,443 bp, encoding a protein of 481 aa. The sequence similarity between IaAO1 and CYP716AL1 was high, being 82%. Therefore, IaAO1 is very likely a triterpene C-28 oxidase. A similar process was applied to screen for triterpene-related UGT candidates. A putative UGT gene (*IaAU*1) was closely clustered with the C-28 glycosyltransferase UGT74M1 from *Saponaria vaccaria* (Figure 2B). *IaAU*1 contains an open reading frame of 1,368 bp, encoding a 459 aa protein with a predicted molecular mass of 50.5 kDa. In addition, IaAU1 contains the plant secondary product glycosyltransferase (PSPG) domain and had a 58.75% similarity with UGT74M1. Therefore, it is plausible that IaAU1 encodes a triterpene C-28 glycosyltransferase.

### Functional Characterization of Amyrin C-28 Oxidase IaAO1

**FIGURE 3:**
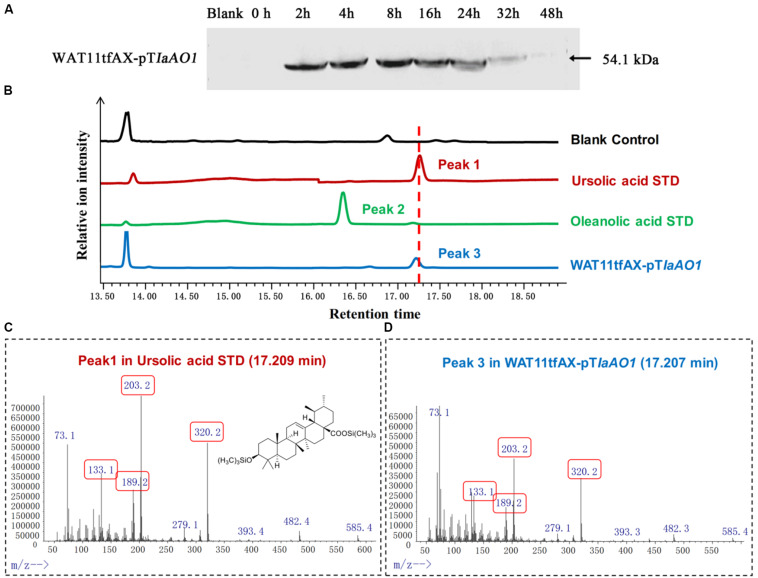
*In vivo* enzymatic activity assays in yeast. **(A)** Western blot analysis of IaAO1 expressed in yeast transformants. Empty vector containing yeast was indicated as blank. **(B)** Total ion chromatograms of ursolic acid (red line), oleanolic acid (green line), WAT11tfAX-pT*IaAO1* (blue line), and blank control WAT11tfAX-pT (black line). Major peaks were numbered. **(C)** Mass spectra of peak 1 from the ursolic acid standard. The chemical structure shown was TMS-derivatized ursolic acid. Characteristic fragment ions were highlighted with red frames. **(D)** Mass spectra of peak 3 from WAT11tfAX-pT*IaAO1*.

In order to elucidate the function of *IaAO*1, it was cloned and expressed in the α-amyrin-producing yeast strain WAT11tfAX. Western blot analysis of the total protein extracted from the cells showed that *IaAO*1 was successfully expressed after induction with 2% galactose (Figure 3A).

Culture extracts from the WAT11tfAX expressing *IaAO*1 yeast were derivatized with trimethylsilylating agents and submitted to GC-MS analysis (Figure 3B). Authentic ursolic acid showed a dominant peak at 17.25 min. A peak with the corresponding retention time was observed in the total ion chromatogram of WAT11tfAX expressing *IaAO*1 yeast, and mass spectra confirmed it was ursolic acid (Figures 3C,D). These results demonstrate that IaAO1 from *I. asprella* catalyzes oxidation at the C-28 position of α-amyrin to yield ursolic acid. The sequence data of *IaAO*1 have been submitted to GenBank with the accession number of MK994507.

### *In vitro* Functional Characterization of C-28 Glycosyltransferase IaAU1

**FIGURE 4:**
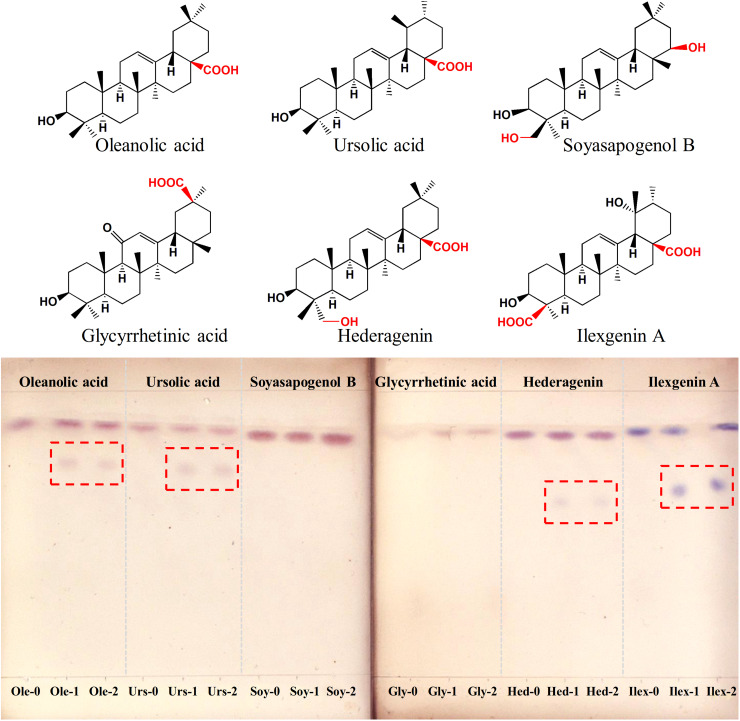
Thin layer chromatograms of IaAU1 assay products of six different sapogenins. “-1” and “-2” represented two replicates of enzyme assay while “-0” suffix indicated reaction with inactivated protein. Assay products were framed in red.

To determine the function of *IaAU*1, the gene was expressed in *E. coli* Rosetta (DE3). SDS-PAGE analysis of total protein, soluble protein, and the purified protein showed a unique band at 68 kDa corresponding to the predicted size of recombinant protein (618 amino acids containing multiple tags from vector) (Supplementary Figure S1). Enzyme activity assays were carried out with the sugar donor UDP-Glc and six triterpene sapogenins, respectively. Primary detection using TLC showed new products were formed with ursane-type triterpenoids (ursolic acid, ilexgenin A) and oleanane-type ones (oleanolic acid, hederagenin), but not with oleanane-type ones without a carboxyl group at C-28 position (glycyrrhetic acid and soyasapogenol B) (Figure 4). The exact mass to charge ratio of assay product was consistent with the expected monoglucosylation molecular ion, i.e., *m/z* 641.4023 (C_36_H_58_O_8_Na^+^, [Urs-Glc]), *m/z* 641.4014 (C_36_H_58_O_8_Na^+^, [Ole-Glc]), *m/z* 657.3968 (C_36_H_58_O_9_Na^+^, [Hed-Glc]), *m/z* 687.3724 (C_36_H_56_O_11_Na^+^, [Ilex-Glc]) (Supplementary Figure S2). Therefore, IaAU1 is deduced to be a glycosyltransferase that can transfer a glucosyl group to the C-28 carboxyl moiety of ursane- or oleanane-type triterpene acids to produce an ester.

**FIGURE 5:**
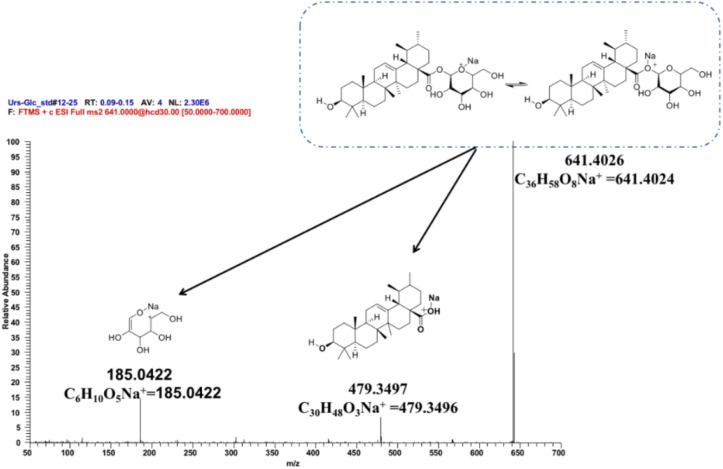
Mass spectra of IaAU1 assay product of ursolic acid. Chemical structures of monoglycoside (*m/z* 641.4026), aglycone (*m/z* 479.3497) and sugar (*m/z* 185.0522) were shown upon the corresponding MS fragments.

To further ascertain the regioselectivity of IaAU1, the product derived from ursolic acid was chosen for structural elucidation. It was isolated in preparative amounts and subjected to MS/MS, and ^1^H- and ^13^C-NMR analyses (Figure 5, Supplementary Figure S3, and Table S3). The MS/MS spectrum clearly demonstrated the characteristic fragment ions of ursolic saponin, namely an aglycone at *m/z* 479.3497 (C_30_H_48_O_3_Na^+^) and a sugar at *m/z* 185.0422 (C_6_H_10_O_5_Na^+^). Compared to the parent compound, the NMR spectra of the product showed additional signals for a glucose moiety, especially δ_H_5.36 (1H, *d*, *J* = 8.0Hz) and δ_C_95.3, indicating the substitution of a glucose at position C-28. As a result, the product was determined to be ursolic acid 28-*O*-β-D-glucopyranoside, which confirmed the proposed function of IaAU1. IaAU1 therefore catalyzes the glycosylation of ursane- or oleanane-type triterpene acids at the C-28 position and has been designated as UGT74AG5 by the UDP-glycosyltransferase (UGT) Nomenclature Committee^3^. The sequence data of *IaAU*1 can be accessed in GenBank under the accession number MK994508.

### Co-expression of *IaAO*1 and *IaAU*1 and Production of Ursolic Acid 28-*O*-β-D-Glucopyranoside

**FIGURE 6:**
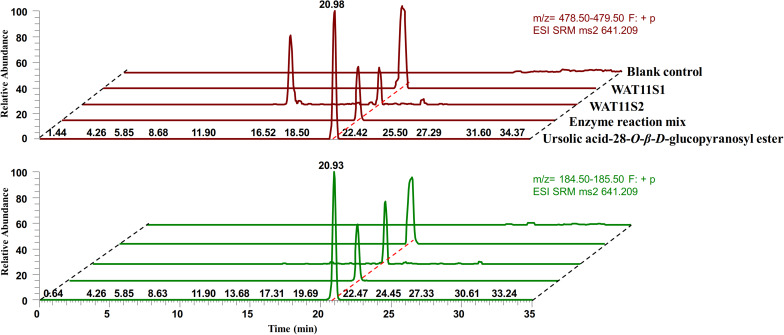
LC-MS/MS SRM mode analysis of ursolic acid 28-*O*-β-D-glucopyranoside. Purified ursolic acid 28-*O*-β-D-glucopyranoside together with the crude enzyme reaction mixture of IaAU1 was used as control. SRM chromatograms of characteristic transitions *m*/*z* 641.2→*m/z* 479.0 was colored in red while *m*/*z* 641.2→*m/z* 185.0 was in green.

To confirm the activity of *IaAO*1 and *IaAU*1, both genes were expressed simultaneously in *S. cerevisiae* WAT11tfAX and the metabolites were analyzed to look for the expected product ursolic acid 28-*O*-β-D-glucopyranoside. Two different yeast strains (WAT11S1 and WAT11S2) were constructed by either transformation of two separate expression plasmids or by integration of two genes into the genome. Analysis of yeast metabolites by LC-MS/MS in selected reaction monitoring (SRM) mode revealed two characteristic transitions (*m/z* 641.2 [Urs-Glc + Na]^+^ →m/z 479.0 [Urs + Na]^+^, *m/z* 641.2 [Urs-Glc + Na]^+^ → *m/z* 185.0 [Glc + Na-H_2_O]^+^) that were clearly present in both of the engineered strains, which was consistent with assay product ursolic acid 28-*O-*β-D-glucopyranoside (Figure 6). However, while WAT11S1 produced up to 27.4 →g/L of Urs-Glc, only a trace amount of Urs-Glc (0.5 →g/L) was detected in WAT11S2. In addition, an unknown peak was observed in the transition *m*/*z* 641.2→*m*/*z* 479.0 of WAT11S2 at about 14 min, indicating the production of some other unexpected compound.

## Discussion

CYPs and UGTs have been demonstrated to be two classes of key enzymes responsible for structural diversity in their biosynthesis. In this study, we identified a cytochrome P450 IaAO1 and a glycosyltransferase UGT74AG5 from *I. asprella*. The biochemical functions of IaAO1 and UGT74AG5 indicated the possible involvement of both genes in the biosynthesis of ursane-type triterpenoids and triterpenoid saponins.

Phylogenetically, IaAO1 belongs to the CYP716A subfamily. Most of the characterized CYP716As catalyze the C-28 oxidation of pentacyclic triterpene scaffolds and a large number of them show activity on multiple substrates (Miettinen et al., 2017). IaAO1 was proven to be an ordinary CYP716A member, catalyzing the oxidation of α-amyrin at C-28. But its substrate specificity has not been investigated herein. Very recently, we isolated the gene encoding IpAO1 (an ortholog of CYP716A210, D. Nelson, personal communication, July 11, 2018) from *I. pubescens*, which has been shown to catalyze the oxidation of both α- and β-amyrin to give ursolic acid and oleanolic acid, respectively (Qin et al., 2019). Since IaAO1 shares 98% sequence identity and 99% sequence similarity with IpAO1, IaAO1 should also be an ortholog of CYP716A210 and must also catalyze the oxidation of β-amyrin at C-28. In accordance, IaAO1 was named as IaCYP716A210.

Glycosylation commonly occurs at the C-3 or C-28 sites in many bioactive pentacyclic triterpene skeletons. To date, only a handful of triterpene UGTs that modify the carboxyl group at C-28 have been characterized, such as UGT73AD1 from *Centella asiatica* (de Costa et al., 2017), UGT73F3 from *Medicago truncatula* (Naoumkina et al., 2010), UGT74M1 from *Saponaria vaccaria* (Meesapyodsuk et al., 2007), UGT73C12 and UGT73C13 from *Barbarea vulgaris* (Augustin et al., 2012). The newly discovered UGT74AG5 provides insight into glycosylation of pentacyclic triterpenoid saponin biosynthesis. An *in vitro* enzymatic assay implied that UGT74AG5 has low substrate specificity but high regiospecificity, which has also been observed with many other UGTs (Rahimi et al., 2019). UGT74AG5 can catalyze the conversion of various ursane-type and oleanane-type carboxylic acids, but it cannot accept substrates without a carboxyl group at C-28. Moreover, MS and NMR analyses of the enzyme product confirmed the substitution of glucose at C-28 in the β-configuration.

**FIGURE 7:**
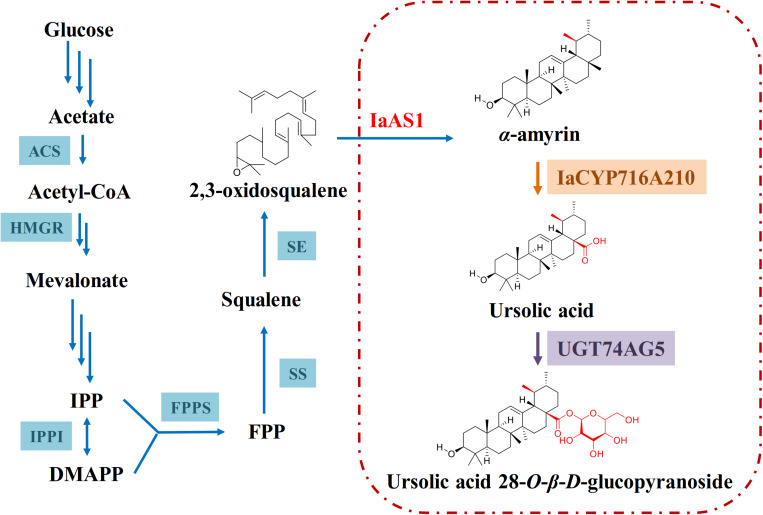
Proposed biosynthetic pathway of ursolic acid 28-*O*-β-D-glucopyranoside in engineered yeast. The triterpene backbone α-amyrin is modified by IaCYP716A210 before ultimately being glycosylated by UGT74AG5.

After functional characterization of IaCYP716A210 and UGT74AG5, they were co-expressed in a yeast strain that had been pre-transformed with the mixed amyrin synthase IaAS1, which led to the production of ursolic acid 28-*O*-β-D-glucopyranoside although in low amounts (Figure 7). Interestingly, this compound has only been found in *Lycopus lucidus var. hirtus* (Li et al., 2014). It has not been isolated from *I. asprella* (Du et al., 2017) or many other flowering plants. This raises the questions as to if ursolic acid is indeed the natural substrate of UGT74AG5 or if ursolic acid 28-*O*-β-D-glucopyranoside is a biosynthetic intermediate and subject to further structural modification in the plant cell. To answer these questions, further studies should be carried out.

## Data Availability Statement

The sequences of both genes identified in this study can be retrieved in GenBank with the accession Nos. MK994507 and MK994508.

## Author Contributions

HX and XJ designed the experiment. XJ and HX wrote the manuscript. XJ, SL, YC, and TW performed the major experiments including gene cloning, enzyme expression and functional characterization. JL contributed to the NMR analysis. ZZ provided the substrate ilexgenin A and helpful guidance. KW, XY, JQ, and CL constructed the yeast strain WAT11tfAX. HX and RZ supervised the entire project. All authors have read and approved the manuscript.

## Conflict of Interest

The authors declare that the research was conducted in the absence of any commercial or financial relationships that could be construed as a potential conflict of interest.

## Footnotes

1 https://www.ncbi.nlm.nih.gov/nuccore/

2 https://www.ebi.ac.uk/Tools/msa/clustalo/

3 https://prime.vetmed.wsu.edu/resources/udp-glucuronsyltransferase-homepage
