# Supplementary material for: Identification of α-Amyrin 28-Carboxylase and Glycosyltransferase From Ilex asprella and Production of Ursolic Acid 28-O-β-D-Glucopyranoside in Engineered Yeast
Source: Front Plant Sci. 2020 May 20;11:612. doi: 10.3389/fpls.2020.00612 (PMC7251064; doi:10.3389/fpls.2020.00612)
Supplement: Supplementary file 1 [file Data_Sheet_1.PDF]

Accession Numbers

Amino acid sequences used for phylogenetic analysis can be found in the GenBank database under the following accession numbers: CYP716A52v2 (AFO63032); CYP716A83 (AOG74832); CYP716AL1 (AEX07773); **IaAO1** (QHB93527); CYP716A75 (AHF22088); CYP716A265 (AZS32332); CYP716A12 (ABC59076); CYP716A266 (AZS32333); CYP716A15 (F6H9N6); CYP716A17 (BAJ84107); CYP716A14v2 (AHF22083); CYP716A1 (AED94045); CYP716A2 (AED94048); CYP716Y1 (AHF45909); CYP87D16 (AHF22090); CYP88D6 (QBC36431); CYP51H10 (ABG88965); CYP93E5 (AIN25417); CYP93E6 (AIN25418); CYP93E7 (AIN25419); CYP93E8 (AIN25420); CYP93E2 (ABC59085); CYP93E3 (BAG68930); CYP93E4 (AIN25416); CYP93E1 (BAE94181); CYP93E9 (AIN25421); CYP72A68v2 (BAL45204); CYP72A61v2 (BAL45199); CYP72A154 (BAL45207); CYP72A63 (CYP72A63); CYP106A1 (ADF38708); UGT73F4 (BAM29363); UGT73F2 (BAM29362); UGT73F3 (ACT34898); UGT73P2 (BAI99584); UGT73P12 (BBN60804); GuUGAT (ANJ03631); UGT73AD1 (ALD84259); UGT73AH1 (AUR26623); UGT73C12 (AFN26668); UGT73C13 (AFN26669); UGT73C10 (AFN26666); UGT73C11 (AFN26667); UGTPg100 (AKQ76388); UGT91H4 (BAI99585); UGTPg29 (AKA44579); UGT74AC1 (AEM42999); **IaAU1** (QHB93528); UGT74M1 (ABK76266); UGT74AE2 (AGR44631); UGTPg45 (AKA44586); Bs-Yjic (NP_389104).

TABLE S1 Primers used in this study

Primer ID	(Primer sequence) 5'→3'	Remarks
IaAO1-F	CATGGAGTTCTTCTATGTCT	Amplification of *IaAO*1
IaAO1-R	TTAAGCTGCTGCTTTGTGCGG	
IaAU1-F	ATGGAGAAAGAAAAAGCTTGCA	Amplification of *IaAU*1
IaAU1-R	TCAGTTGGCCACAAGCCG	
pTIaAO1-F	TTGAAAATTCGAATTCATGGAGTTCTTCTATGTCTCTCTCCTCTCTCTCTTCG	Amplification of *IaAO*1 for ligation into pESC-TRP
pTIaAO1-R	GAATTGTTAATTAAGAGCTCTTA***ATGATGATGATGATGATG***AGCTGCTGCTTTGTGCGG	
pETIaAU1-F	GCCATGGCTGATATCATGGAGAAAGAAAAAGCTTGCAAAGC	Amplification of *IaAU*1 for ligation into pET32a(+)
pETIaAU1-R	TTGTCGACGGAGCTCTCAGTTGGCCACAAGCCGAGC	
pUIaAU1-F	TTGAAAATTCGAATTCATGGAGAAAGAAAAAGCTTGCAAAGC	Amplification of *IaAU*1 for ligation into pESC-URA
pUIaAU1-R	GAATTGTTAATTAAGAGCTCTCAGTTGGCCACAAGCCGAGC	
GPDIaAO1-F	TAGAACTAGTGGATCCATGGAGTTCTTCTATGTCTCTCTCCTCT	Amplification of *IaAO1* for ligation into p426GDP
GPDIaAO1-R	AATTACATGACTCGAGTTAAGCTGCTGCTTTGTGCGGA	
GPDIaAU1-F	CGGATTCTAGAACTAGTGGATCCATGGAAAAGGAAAAGGCATGCAAAG	Amplification of *IaAU1* for ligation into p426GDP
GPDIaAU1-R	CATAACTAATTACATGACTCGAGTTAATTTGCTACCAATCTGGCAACGATTTCAT	
ADE2-GAP-F	CATCCTACTATAACAATCAAGAAAAACAAGAAAATCGGACAAAACAATCAAGTGGGAAC AAAAGCTGGAGCTCAGTTT	Amplification of P_{GPD}-*IaAO1*-T_{CYC1} expression cassette for insertion into ADE2 site of yeast genome
ADE2-CYC1-R	GTATATCATTTTATAATTATTTGCTGTACAAGTATATCAATAAACTTATATAGGCCGCAAA TTAAAGCCTTCGAGCGTCC	
BTS1-GAP-F	GAGGAGAGAAGGCTTTATTTCTGACTATCTTCCTCCACTAATTTGATTGATCAATTTATTTC ATTATCAATACTCGCCATTTCAAAG	Amplification of P_{GPD}-*IaAU1*-T_{CYC1} expression cassette for insertion into BTS1 site of yeast genome
BTS1-CYC1-R	TCATTTTCAAAGAAGCTACTAATAGAAAGAGAACAAAGCGTTTACGAGTCTGGAAAATCA GCAAATTAAAGCCTTCGAGC	

Note: "__" stands for homology extent of each end at insert site. The italicized parts indicate 6×HIS tags.

TABLE S2 Plasmids and strains used in this study

Plasmid or strain	Description or relevant genotype	Source or reference
**Plasmids**		
pEASY-T5	Cloning vector with a T7 promoter, Amp^r, Kan^r	TransGen Biotech
pET-32a(+)	Bacterial vector for expressing fusion proteins with His-, Trx- and S-tag, Amp^r	Novagen
pESC-TRP	Galactose-regulated yeast expression vector with a TRP1 selectable marker, Amp^r	Lab stock
pESC-URA	Galactose-regulated yeast expression vector with a URA3 selectable marker, Amp^r	Lab stock
p426GPD	Yeast expression vector with a GAP promoter, Amp^r	ATCC
Cas9-NAT	The vector with a natMX6 yeast selectable marker for expressing Cas9 protein, Amp^r	Addgene
pRS42H-gRNA-*ade*2	The vector carrying *ade*2 guide RNA of *S. cerevisiae*	Constructed by Yun
pRS42H-gRNA-*bts*1	The vector carrying *bts*1 guide RNA of *S. cerevisiae*	(unpublished data)
*p*T5-*IaAO1*	Coding region of *IaAO1* cloned into *pEASY*-T5, Amp^r, Kan^r	This study
*p*T5-*IaAU1*	Coding region of *IaAU1* cloned into *pEASY*-T5, Amp^r, Kan^r	This study
pET-*IaAU1*	Coding region of *IaAU1* cloned into the *EcoR* V-*Sac* I sites of pET-32a(+),Amp^r	This study
pT-*IaAO1*	Coding region of *IaAO1* cloned into the *EcoR* I-*Spe* I sites of pESC-TRP, Amp^r	This study
pU-*IaAU1*	Coding region of *IaAU1* cloned into the *EcoR* I-*Sac* I sites of pESC-URA, Amp^r	This study
GPD-*IaAO1*	Coding region of *IaAO1* cloned into the *BamH* I-*Xho* I sites of Ip426GPD, Amp^r	This study
GPD-*IaAU1*	Coding region of *IaAU1* cloned into the *BamH* I-*Xho* I sites of p426GPD, Amp^r	This study
**Strains**		
***E. coli* strains**		
*Trans*1-T1	F$^-$ φ80(*lac*Z)△M15△*lac*X74*hsd*R(r_k^-, m_k^+)△*rec*A1398*end*A1*ton*A	TransGen Biotech
*Trans*etta (DE3)	F$^-$ *omp*T *hsd*S$_B$($r_B^-m_B^-$)*gal dcm lac*Y1(DE3)pRARE(argU, argW, ileX, glyT, leuW, proL)(Cam^r)	TransGen Biotech
***S. cerevisiae* strains**		
WAT11tfAX	WAT11*, *trp*1::P_{GAP1}-*SctHMGR1*-T_{CYC1}, *ura*3::P_{GAP1}-*ScERG20*-T_{CYC1}, *leu*2::P_{GAP1}-*SeACS*^{L641P}-T_{CYC1}, *his*3::P_{TEF1}-*IaAS1*-T_{CYC1}	Constructed by Yun (unpublished data)
WAT11tfAX-pT*IaAO1*	WAT11tfAX carrying pT*IaAO1* plasmid	This study
WAT11tfAX-pT	WAT11tfAX carrying pESC-TRP plasmid	This study
WAT11tfAX-pT-pU	WAT11tfAX carrying both pESC-TRP and pESC-URA plasmids	This study
WAT11S1	WAT11tfAX carrying both pT*IaAO1* and pU-*IaAU1* plasmids	This study
WAT11tfAX-Cas9	WAT11tfAX carrying Cas-NAT plasmid for construction of WAT11S2	This study
WAT11S2	WAT11tfAX, *ade2*::P_{GAP}-*IaAO1*-T_{CYC1}, *bts*1::P_{GAP}-*IaAU1*-T_{CYC1}	This study

*Urban P, Mignotte C, Kazmaier M, Delorme F, Pompon D. 1997. Cloning, yeast expression, and characterization of the coupling of two distantly related *Arabidopsis thaliana* NADPH-cytochrome P450 reductases with P450 CYP73A5*. Journal of Biological Chemistry. 272(31);19176-19186. doi: 10.1074/jbc.272.31.19176

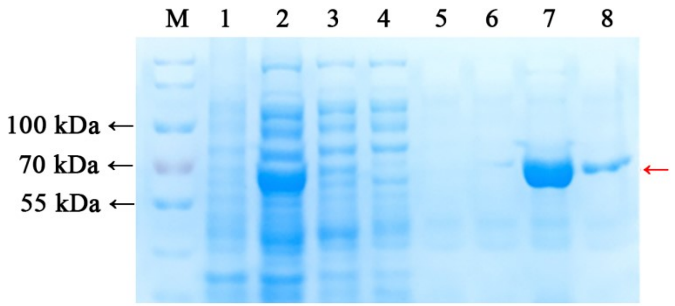

FIGURE S1 Expression and purification of recombinant protein IaAU1. M, molecular mass standard; 1, total protein before induction; 2, total protein after induction; 3, soluble protein; 4, flow-through; 5-8, purified IaAU1.

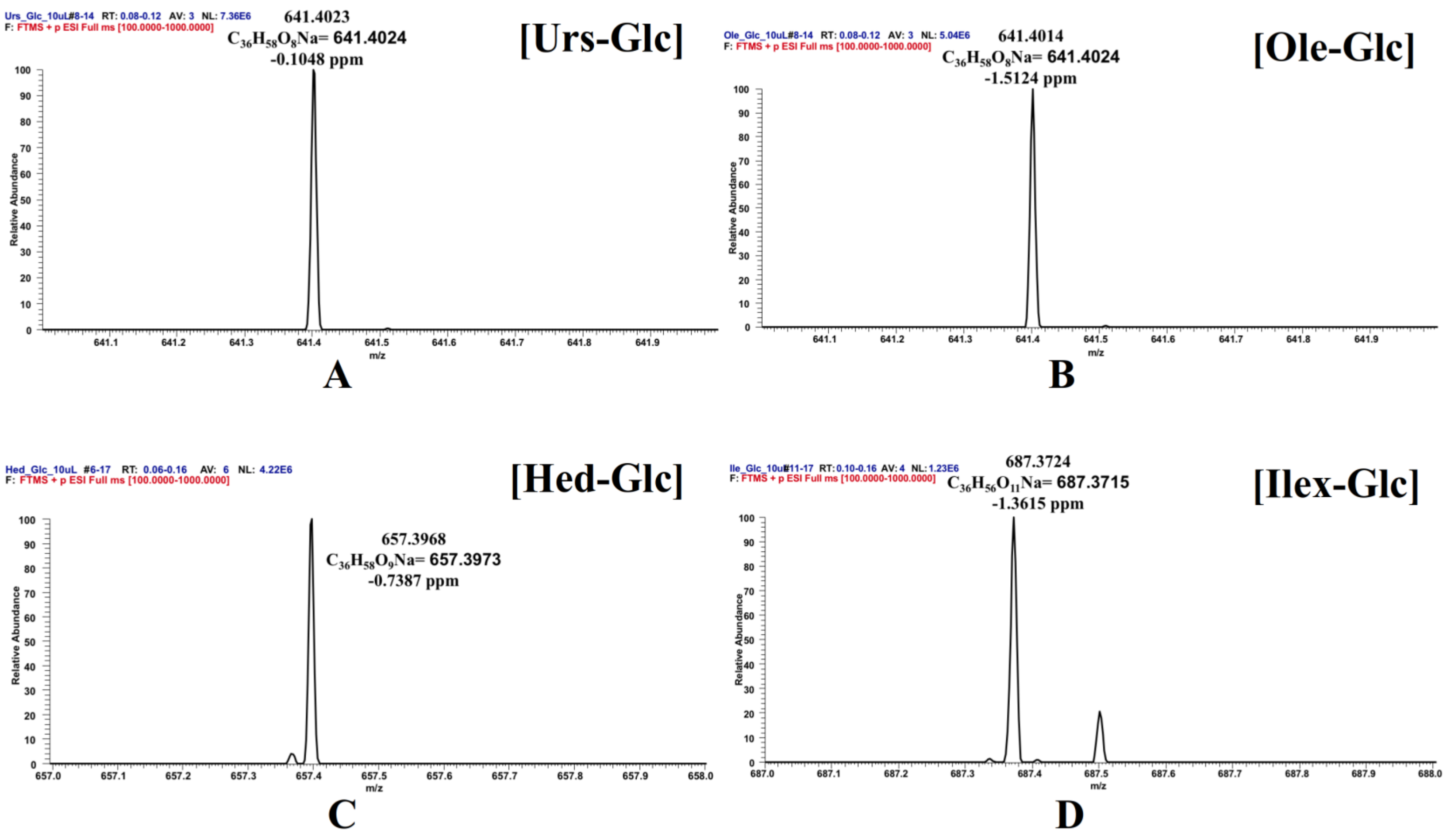

FIGURE S2 Mass to charge ratio of IaAU1 assay product of (A) ursolic acid; (B) oleanolic acid; (C) hederagenin; (D) ilexgenin A.

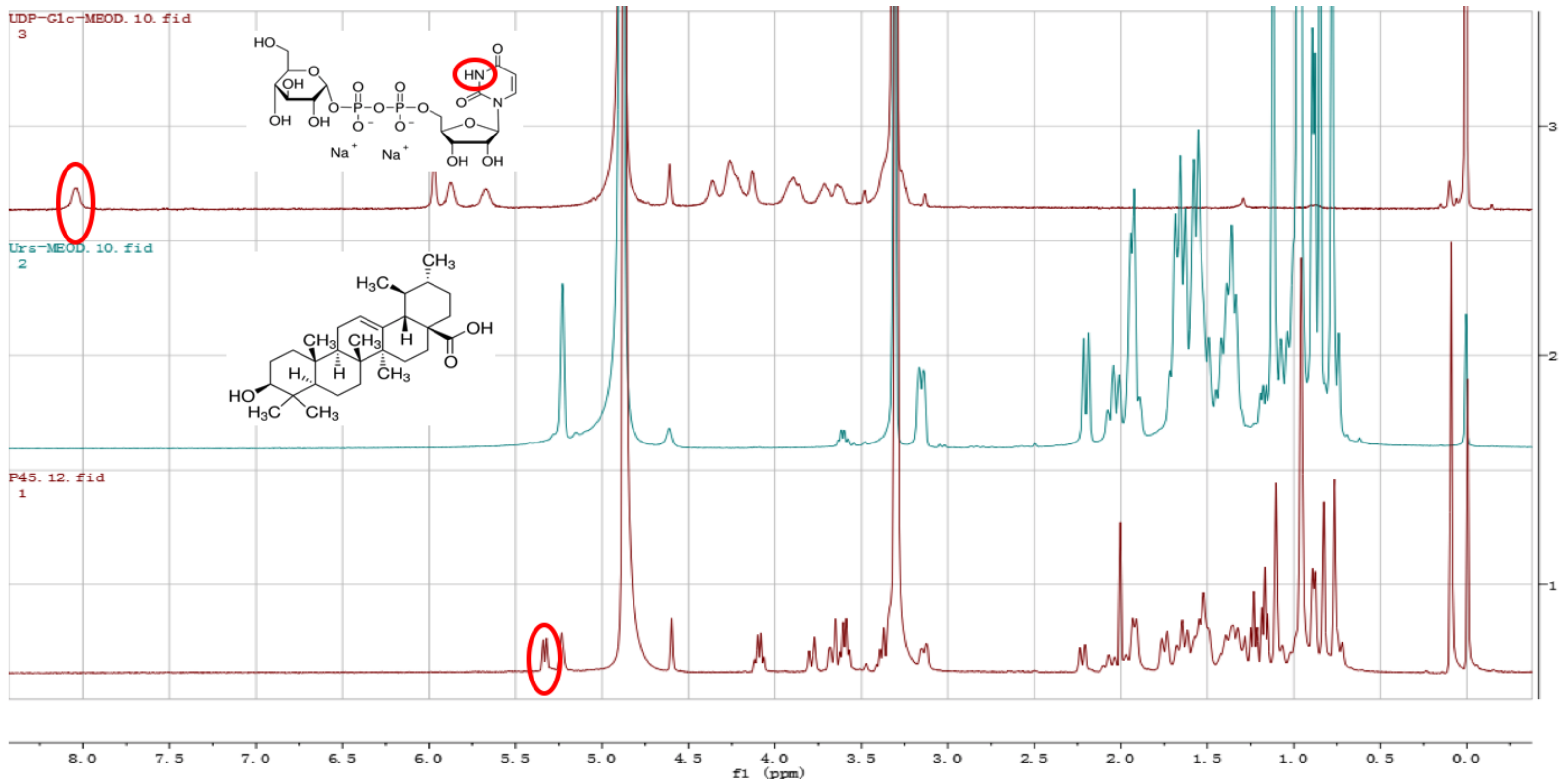

FIGURE S3 ^{1}H NMR analysis of UDP-glucose (above), ursolic acid (middle) and enzymatic reaction product (below)

TABLE S3 ^{1}C-NMR data for ursolic acid and enzymatic reaction product

Position of carboxyl	Ursolic acid	Reaction product	Position of carboxyl	Ursolic acid	Reaction product
1	39.0	39.9	19	38.4	39.4
2	27.8	28.9	20	38.6	31.3
3	78.3	79.3	21	30.4	28.4
4	38.6	39.7	22	38.4	37.1
5	55.4	56.4	23	22.7	28.4
6	20.2	19.1	24	16.4	16.5
7	33.0	33.9	25	16.2	15.7
8	39.0	40.60	26	18.1	17.5
9	-	-	27	23.9	23.6
10	36.7	37.7	28	180.2	177.5
11	23.0	24.0	29	20.2	17.2
12	125.5	126.8	30	18.1	21.1
13	138.2	138.7	1'	-	95.3
14	41.9	42.9	2'	-	73.5
15	27.83	28.89	3'	-	79.33
16	23.94	24.84	4'	-	70.81
17	-	-	5'	-	78.16
18	52.99	53.82	6'	-	62.11
